# Embryonic lethality leads to hybrid male inviability in hybrids between *Drosophila melanogaster* and *D. santomea*

**DOI:** 10.1002/ece3.573

**Published:** 2013-04-23

**Authors:** Jackie Gavin-Smyth, Daniel R Matute

**Affiliations:** 1 Ecology and Evolution, The University of Chicago920 East 58th Street, Chicago, Illinois, 60637, USA; 2 The Chicago Fellows Program, The University of Chicago920 East 58th Street, Chicago, Illinois, 60637, USA; 3 Department of Human Genetics, The University of Chicago920 East 58th Street, Chicago, Illinois, 60637, USA

**Keywords:** *Drosophila*, embryonic lethality, Haldane's rule, postzygotic isolation

## Abstract

The study of the morphological defects unique to interspecific hybrids can reveal which developmental pathways have diverged between species. *Drosophila melanogaster* and *D. santomea* diverged more than 10 million years ago, and when crossed produce sterile adult females. Adult hybrid males are absent from all interspecific crosses. We aimed to determine the fate of these hybrid males. To do so, we tracked the development of hybrid females and males using classic genetic markers and techniques. We found that hybrid males die predominantly as embryos with severe segment-specification defects while a large proportion of hybrid females embryos hatch and survive to adulthood. In particular, we show that most male embryos show a characteristic abdominal ablation phenotype, not observed in either parental species. This suggests that sex-specific embryonic developmental defects eliminate hybrid males in this interspecific cross. The study of the developmental abnormalities that occur in hybrids can lead to the understanding of cryptic molecular divergence between species sharing a conserved body plan.

## Introduction

The range of developmental and morphological defects seen in interspecific hybrids spans from complete hybrid lethality to a mild perturbation of adult morphology (reviewed in Coyne and Orr 2004). If any one of these defects has a fitness effect and/or precludes the possibility of gene flow between the parental species, then they will also contribute to reproductive isolation. Detailed study of the developmental defects unique to interspecific hybrids can reveal which traits can serve to keep species apart though postzygotic isolation. While the developmental defects of interspecific hybrids are widespread taxonomically (Coyne and Orr 2004), the specific genetic mechanisms involved are understood in only a few notable examples (reviewed in Nosil and Schluter 2011; Maheshwari and Barbash 2011). Nonetheless, postzygotic isolation follows several patterns that can be generalized to a wide variety of taxonomic groups.

A common pattern of postzygotic isolation is Haldane's rule (Haldane 1922; Orr 1997): in hybridizing species with sex chromosomes, if one sex suffers hybrid breakdown it is usually the heterogametic sex. In *Drosophila* and mammals, it is predominantly the male XY hybrids that suffer the most pronounced inviability or sterility, while in Lepidopterans and birds, the ZW female hybrids are more likely to be unfit (Orr 1997; Presgraves and Orr 1998; Price 2008). One simple explanation of this pattern is that the majority of genes involved in hybrid inviability are recessive. The hemizygosity of the sex chromosomes in the heterogametic sex, then, allows for full expression of the alleles regardless of their dominance, and their deleterious fitness effects will be more pronounced than in the homogametic individuals (Original formulation in Muller 1940; corrected in Orr 1993a, b; Orr and Turelli 1996).

Instances of Haldane's rule are widespread, however, detailed characterization of the deleterious phenotypes is rare and the genetic basis of heterogametic hybrid inviability has been defined only in select cases. This is due in part to the difficulties of studying the causative genetic differences in interspecific hybrids, especially those resulting in inviability. Nonetheless, a series of efforts have been successful in identifying the genomic regions underlying male lethality and sterility in interspecific *Drosophila* hybrids and many of the specific genes involved (Sawamura et al. 1993a, b; Hutter and Ashburner 1987; Sawamura and Yamamoto 1997; Presgraves et al. 2003; Phadnis and Orr 2009; Tang and Presgraves 2009 among others).

The most success in identifying the developmental defects underlying hybrid inviability has been with *Drosophila*. One well-studied example is the hybrids from the cross of *D. melanogaster* females and males from the *D. simulans* species group (*D*. *simulans, D. sechellia,* and *D. mauritiana*, henceforth referred as the *sim* species group). Even though these two groups of species (*D. melanogaster* and the *sim* species group) diverged approximately 3–5 million years ago (Tamura et al. 2004), the cross produces sterile hybrid adult females. The hybrid male larvae grow slowly (Sanchez and Dübendorfer 1983; Bolkan et al. 2007), cannot molt into pupae and eventually die due to profound mitotic defects (Orr et al. 1997; Barbash et al. 2000; Barbash et al. 2003; Bolkan et al. 2007). Although the causative molecular defects in cell division, the arrest in the G phase of mitosis, and consequent larval lethality are seen in all the three kinds of hybrids between *D. melanogaster* females and the males from the *sim* group of species, the precise developmental consequences of these mitotic defects differ between hybrids. In the *D. melanogaster*/*D. simulans* cross, male larvae usually lack imaginal discs (Seiler and Nothiger 1974; Sanchez and Dübendorfer 1983; Orr et al. 1997). On the other hand, the *D. melanogaster*/*D. mauritiana* cross produces hybrid male larvae that possess imaginal discs, however, a substantial fraction of discs are underdeveloped and threadlike (Sanchez and Dübendorfer 1983; Orr et al. 1997).

**Figure 1:**
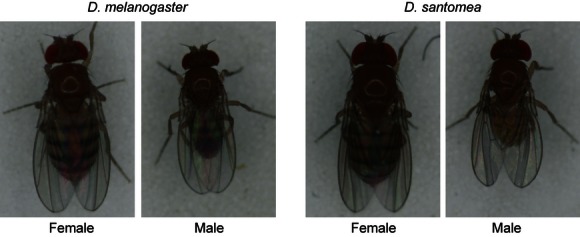
*Drosophila melanogaster* and *Drosophila santomea*. *D. melanogaster* is a cosmopolitan species found in every continent (but Antarctica). *D. santomea*, on the other hand, is an endemic species whose geographic range is restricted to the rainforests in the mountains of São Tomé.

With the exception of these examples in the *simulans* group, very little is known about the developmental processes disrupted in hybrid males of other interspecific crosses between *Drosophila*. For example, it is known that more divergent species (*D. simulans* × *D. teissieri*, *D. melanogaster* × *D. santomea*) can produce hybrid progeny but in all cases, the progeny is exclusively sterile females (Orr 1993a, b; Matute et al. 2010). It has yet to be determined whether male lethality in crosses between these more divergent species arises from developmental defects similar to those in the *D. melanogaster*/*sim* group of interspecific hybrids. Here, we examine the developmental stage of male hybrid lethality in crosses of *D. melanogaster* and *D. santomea* (Fig. 1). These two species diverged more than 10 million years ago (Tamura et al. 2004) but recent work has reported that crosses between *D. melanogaster* females and *D. santomea* males produce sterile females (Matute et al. 2010). While the genetic architecture of female inviability in these hybrids has been assessed, male hybrid lethality has not been examined in detail. To date, the *D. yakuba* lineage (*D. yakuba, D. teissieri,* and *D. santomea*) and the *melanogaster* lineage (*D. melanogaster* and the *sim* group of species) remain the most divergent clades in *Drosophila* shown to hybridize (Orr 1993a, b; Matute et al. 2010). The study of developmental defects in hybrids between these divergent clades may help uncover the cryptic molecular variation and the evolution of developmental differences between species with conserved patterning systems and body plans. In this initial approach, we used classic developmental genetics techniques to distinguish and track the development of *D. melanogaster*/*D. santomea* hybrid females and males. We found that hybrid males die predominantly during embryogenesis manifesting severe segment-specification defects, while hybrid females usually hatch and survive to adulthood.

## Materials and Methods

### *Drosophila* stocks

All transgenic *D. melanogaster* lines were obtained from the Bloomington *Drosophila* Stock Center at Indiana University (http://flystocks.bio.indiana.edu/) and are listed in Table S1. *D. santomea* SYN2005 is an outbred line constructed by combining isofemale stocks and kept in large numbers since its initiation (Matute et al. 2009; Matute and Coyne 2010). All the other stocks of *D. santomea* were collected in 2009 by DRM and were established as isofemale lines (i.e., progeny from a single inseminated female has been perpetuated in laboratory conditions).

### Crosses

All flies were raised on standard cornmeal/molasses medium in pint size bottles or 8-dram plastic vials. Virgins females were collected by lightly gassing recently emerged flies (less than 8 h after eclosion) with CO_2_ and separating females from males_._ For embryonic lethality counts and cuticular phenotypes, interspecific crosses were started in vials with at least twenty >3-day-old females and at least 40 *D. santomea* males. (Intraspecific crosses were housed in plastic cups directly after virgin females and males were mixed.) The crosses were incubated in a light-cycling incubator at a constant temperature (25°C) and relative humidity (60%) simulating days of 14 h of light and 10 h of darkness. In all F_1_ hybrids, the identity of the mother is shown first (e.g., *mel*/*san* is the progeny of *D. melanogaster* females and *D. santomea* males).

### Insemination rate

To directly measure the fertilization rates in hybrid crosses, we combined at least 100 *D. melanogaster* females and 200 *D. santomea* males and housed them in the same vials for 4 days. After that time, females were dissected and the spermatheca and oviducts were mounted in Ringer's Solution at 4°C to check for the presence of sperm. If we observed any sperm, even weak or scarce, in the dissected female tract, we categorized that female as mated. The frequency of insemination was established for 32 isofemale lines. Three replicates of independent crosses were measured per isofemale line.

### Embryo collection

After 2–3 days of crossing, both male and female flies were transferred to a collection cup with a standard yeasted apple juice plate to allow oviposition. In all crosses, embryonic lethality was quantified by counting the number of hatched and unhatched fertilized eggs of an overnight deposition after 24 h of aging at 25°C. Briefly, viability was scored as the number of empty egg cases, while lethality was scored as unhatched eggs with discernible larval structures. We follow the same procedure to quantify lethality rates for intraspecific crosses. We quantified lethality for at least three replicates per cross.

For visualization of cuticles of unhatched embryos, we first dechorinated and devitellenized unhatched embryos manually. These embryos were transferred to an emulsion of Acetic acid:Glycerol (3:1) to digest all soft tissues. Digested cuticles were then mounted in Hoyer's Media:85% Lactic Acid (1:1). All slides were then moved overnight to a baking oven (75°C) to speed clearing. Embryos were then imaged and scored for cuticular defects.

For the distinction between male and female larval cuticles, we *crossed y,w; P{Sxl-Pe-EGFP.G}* (Thompson et al. 2004) *D. melanogaster* females to *D. santomea* males. The *y,w; Sxl:GFP* females were generated from stocks from Bloomington stock center (Stock number: 24105; w*; P{Sxl-Pe-EGFP.G}G78b).

Crosses involving *y,w; Sxl:GFP* females were allowed to proceed for 3–4 days in a cornmeal 8-dram vial as described above and then transferred to an apple juice plate. After an overnight deposition, embryos were sorted by GFP expression under a dissecting fluorescent microscope after 6 h of incubation at 25°C. The *SXL::GFP* distinction was additionally checked by observing the color of the mouth parts of hatched larvae or cuticles of dead embryos, as the lack of pigment in the larval mouthparts indicated the presence of a single *yellow* marked X-chromosome from *D. melanogaster*. The *yellow* allele from *D. santomea* rescues the *D. melanogaster yellow* allele's mouthpart phenotype, therefore all the dead embryos or larvae with wild-type mouthparts were considered females.

We also used the lack of pigmentation of *yellow* mouthparts to sex-type the cuticles of those embryos which failed to hatch. We then classified the severity of Anterior–Posterior patterning defects present in each sex with the following metric: The absence of denticulate bands beyond abdominal five, was scored as an “ablation” phenotype or A5-. If cuticle was present, but without discernible denticle bands, embryos were classified as intermediate. Finally, if some amount of denticulate matter, however, slight, was discernible posterior to abdominal segment 5, then the cuticle was scored as A5+.

### Larval and pupal lethality

We collected 50 L1 larvae within 12 h after egg hatching and transferred them to an 8-dram cornmeal plastic vial. Vials were checked daily and once L2 larvae were observed, we damped the food with a 0.5% propionic acid solution and added a pupation substrate to the vial (Mierly Clark, Kimwipes Delicate Task, Roswell, GA). After a few days (seven on average), the number of pupal cases on the paper was counted. (If larvae pupated on the food media, the vial was not taken into account.) The ratio of pupae to L1 larvae was used as a proxy of larval viability. For each vial, we also quantified how many adults hatched and calculated a pupal viability index (i.e., ratio of adult/pupal cases).

### Statistical analyses

All statistical analyses were done with R (R Development Core Team 2005). In three cases (B1300.17, C1350.14, and C1350.15), we were able to collect two replicates for the estimates of hybrid viability. As all the other lines were represented by three replicates, we estimated missing values by calculating the average of the available replicates. Fertilization rate, pure species estimates of lethality, larval and pupal survival rates of hybrid individuals significantly deviated from normality (even after data transformation) and were analyzed using a Kruskal–Wallis test. Lethality rates in hybrid crosses were analyzed with a one-way ANOVA (residuals of the linear model followed a normal distribution; Shapiro–Wilk normality test: *W* = 0.982, *P* = 0.319).

## Results

**Figure 2:**
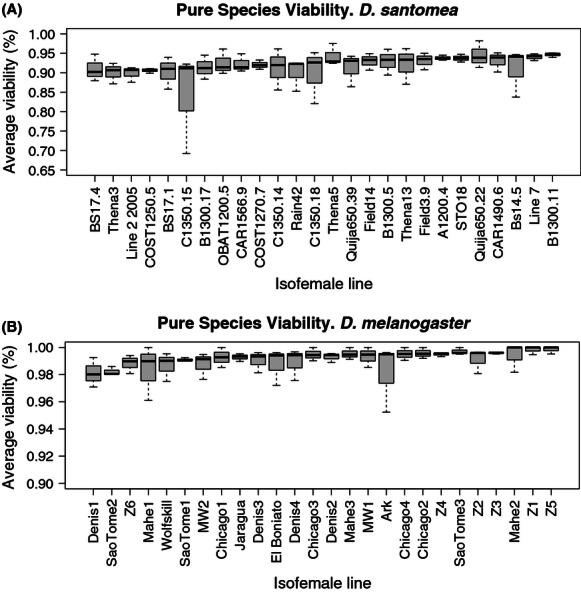
Pure species Viability. Viability rates in pure species in *Drosophila santomea* isofemale lines (A) and *D. melanogaster* (B). For each species, we analyzed 26 isofemale lines (three replicates per line) and found no heterogeneity in viability levels. Isofemale lines were ordinated by their median viability (thick line in the each box). The bottom edge of the box is the 25th percentile of the data and the top edge of the box is the 75th percentile of the data.

To study the nature of postzygotic isolation in *mel*/*san* hybrids, we first analyzed the rates of embryonic lethality in pure species isofemale lines of *D. santomea* and *D. melanogaster*. When measured at 24°C, the lethality rates are lower than 15% in all *D. santomea* isofemale lines and lower than 5% in all the measured *D. melanogaster* isofemale lines (Fig. 2). We detected no significant heterogeneity within the species (*D. santomea*: Kruskal–Wallis *χ*^2^ = 20.619, df = 25, *P* = 0.714; *D. melanogaster*: Kruskal–Wallis *χ*^2^ = 27.242, df = 24, *P* = 0.293), but there were significant differences between species (Mean viability of *D. melanogaster* embryos: 99.12%; Mean viability of *D. santomea* embryos: 91.81%; Wilcoxon rank sum test with continuity correction data: *W* = 6058, *P* < 2.2 × 10^−16^). Additionally, the majority of the individuals from the two parental species show no major developmental defects and both sexes are present at roughly the same frequencies (Fig. 2, Table S2) indicating that no sex-specific lethality was detectable in the parental species. When *D. melanogaster* females are crossed to *D. santomea* males, however, only hybrid females are observed as adults, indicating complete sex-specific lethality in these hybrid crosses. We then sought to determine the efficacy of mating and the developmental defects that lead to hybrid male inviability in this highly divergent cross of *Drosophila* species.

First, we measured the insemination rates when *D. melanogaster* females from the ArkLa outbred line (Matute et al. 2010) were mated to males of different *D. santomea* isofemale lines (*n* = 32 lines, three replicates per line). The proportion of *D. melanogaster* females that accepted *D. santomea* males was assessed by the presence or absence of sperm in their reproductive tract after 4 days of being housed together. We detected marginal hetero-geneity in the proportion of inseminated females (Kruskal–Wallis *χ*^2^ = 44.047, df = 31, *P* = 0.061) suggesting that the level of behavioral isolation of *D. melanogaster* ArkLa females toward males of all lines of *D. santomea* is high with little variation (average proportion of inseminated females per cross: 2.55%, SEM = 2.86 × 10^−3^).

**Figure 3:**
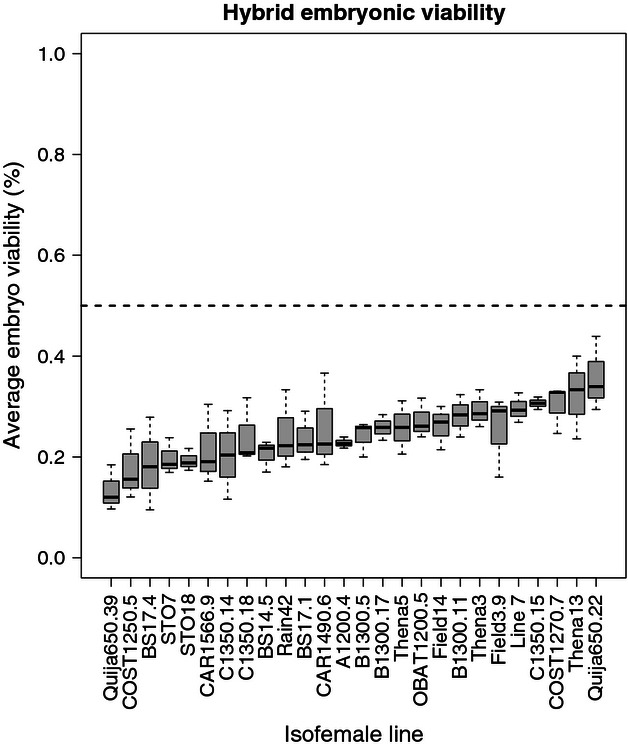
Hybrid embryonic lethality in different *mel*/*san* crosses**.** We measured hybrid embryonic viability for different *Drosophila santomea* lines when crossed to *D. melanogaster* ArkLa females. Isofemale lines were ordinated by their median viability. The dotted line shows the expectation of full male embryonic lethality and no female embryonic lethality. The bottom edge of the box is the 25th percentile of the data and the top edge of the box is the 75th percentile of the data.

We then measured the rate of embryonic lethality in F_1_ hybrids between *D. melanogaster* and *D. santomea* by crossing *D. melanogaster* ArkLa females to males of 26 lines of *D. santomea*. On average, 75% of the hybrid embryos from these crosses were inviable (25% were viable, Fig. 3), although there was significant heterogeneity in viability caused by the effect of the parental line (one-way ANOVA: *F*_25,52_ = 2.266, *P* = 6.48 × 10^−3^). Despite this heterogeneity, the embryonic lethality of hybrid crosses between *D. melanogaster* and *D. santomea* lines was significantly higher than that observed in either of the two pure species (Wilcoxon rank sum test with continuity correction; *W* > 6084, *P* < 2 × 10^−16^).

As over 50% of the hybrid embryos did not hatch into L1 larvae in all crosses, we hypothesized that the majority of those embryos that failed to hatch were the hybrid male progeny. To determine whether there was any sex-biased lethality during hybrid embryogenesis, we repeated the crosses with 11 *D. santomea* lines (10 isofemale lines and *D. santomea* SYN2005). Using two different sex-specific markers, a *SXL:GFP* reporter (Thompson et al. 2004) which is detectable only in females after 4–6 h of embryogenesis and *yellow* which manifests as a recessive marker in the larval mouth hooks, we assessed the sex-specific embryonic lethality rates in the F_1_ hybrids. We found that the vast majority of *SXL::GFP*^-^ (hybrid males) embryos failed to hatch, regardless of the *D. santomea* line (Table 1). Even though a few hybrid male embryos hatched into a L1 larvae, however, L2 male larvae were never observed. These data strongly indicate that the F_1_ male hybrid embryos of *D. melanogaster* and *D. santomea* are rendered inviable during the course of embryogenesis.

**Table 1:** The relative frequency of dead/live embryos in each sex in *Drosophila melanogaster*/*D. santomea* hybrids measured using *sxl::GFP* and *yellow* as sex-specific markers. All lines showed a uniform male embryonic lethality (close to 100%) but there was significant variation in the degree of female inviability

	GFP (+)		GFP (−)			
	
Line	Dead	Alive	Dead	Alive	Surviving females (%)	Surviving males (%)
STO7	46	73	114	1	61.344	0.870
Quija650.39	51	96	154	0	65.306	0
COST1250.5	36	104	134	0	74.286	0
A1200.4	44	140	174	1	76.087	0.571
Field14	20	73	83	0	78.495	0
Bs14.5	30	103	124	1	77.444	0.8
SYN2005	26	90	100	3	77.586	2.913
Thena13	19	74	94	0	79.570	0
Thena3	18	92	102	1	83.636	0.971
COST1270.7	12	67	75	1	84.810	1.316
Quija650.22	5	58	59	2	92.0635	3.279

In contrast to the nearly complete male embryonic lethality, in all crosses a significant proportion (>50%) of the hybrid females survived embryogenesis (Table 1). The sex-specific viability counts indicate that there was significant variation in the frequency of viable females (Kruskal–Wallis *χ*^2^ = 26.028, df = 10, *P* = 3.70 × 10^−3^) but not of male lethality rates (male larvae were observed only in nine out of the 33 replicates). The significant differences in female lethality suggest that there are genetic variants in the paternal genome with differential effects on the embryonic viability of hybrid females.

**Figure 4:**
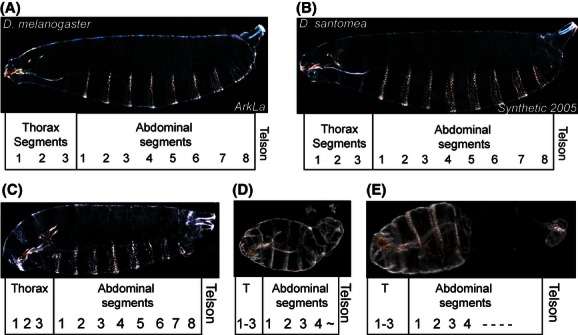
Typical cuticles manifesting a “complete” “intermediate” or “ablation” phenotype. Darkfield micrographs of wild-type pure species larval cuticles of *Drosophila melanogaster* (A) and *D. santomea* (B) with the major thoracic and abdominal segments mapped below. (C–D) Hybrid larval cuticles typifying the three major categories of defects in those embryos that failed to hatch.

**Figure 5:**
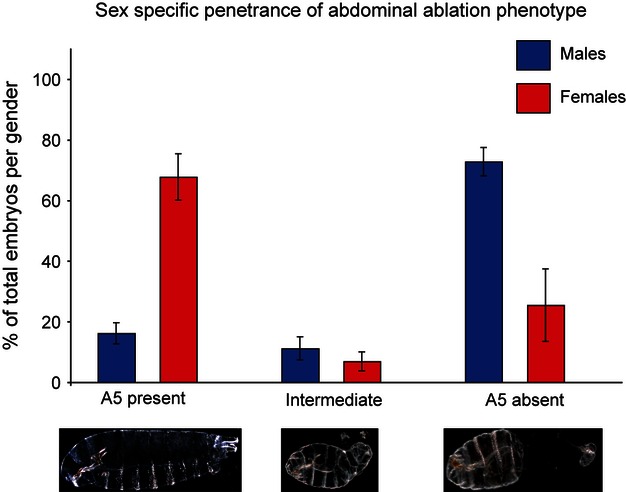
The ablation phenotype is disproportionally seen in hybrid *mel*/*san* males. Three kinds of developmental defects observed in *mel*/*san* hybrids ordinated by increasing severity from right to left. Approximately 67% of hybrid male cuticles manifest abdominal ablations in segments A5 to A7 while >30% females show the same developmental defect. Red bars: females; Blue bars: males. Error bars show the SEM across isofemale lines (see text for details).

To determine whether male hybrid embryos fail to hatch due to a gross sex-specific patterning defect during embryogenesis, we then examined the cuticles of inviable embryos. The male embryos that failed to hatch had a higher prevalence of an abdominal ablation phenotype of the larval cuticle (Figs. 4–5), while the majority of the inviable embryos of both sexes have head involution defects (Fig. S1). Abdominal defects were more frequent in hybrid males than in hybrid females (Two-sided Fisher's Exact Test for Count Data, in all three assayed lines *P* < 0.010, Fig. 5)**.** We conclude, therefore that, at high frequency, male hybrids carrying the *D. melanogaster* X*-*chromosome are unable to pattern the posterior abdominal segments (A5-A7). As no known mutant within *D. melanogaster* recapitulates the developmental defect we observed in these hybrid males, we believe this is a functional antimorphism unique to the hybrid embryo context. This developmental defect is rescued in some hybrid females, possibly due to the presence of the *D. santomea* X-chromosome, as ∼70% of dead *D. melanogaster*/*D. santomea* SYN2005 female hybrids embryos show intact posterior segments.

We followed the development of the female hybrids that survive embryogenesis during their larval and pupal development to establish whether there was any hybrid inviability during these later developmental stages. Table 2 shows the overall high survival rate of hybrid females during larval and pupal stages. These results suggest that the majority of mechanisms that cause hybrid incompatibility in both males and females are active primarily during embryogenesis. After the embryonic stage, hybrid females have comparable survival rates to the parental species, with little heterogeneity between lines; Kruskal–Wallis rank sum test, Larval viability: Kruskal–Wallis *χ*^2^ = 19.577, df = 25, *P* = 0.769; Pupal viability: Kruskal–Wallis *χ*^2^ = 34.632, df = 25, *P* = 0.095). Thus, genetic variation in the *D. santomea* population gives rise to differential penetrance of embryonic patterning defects, but has little effect on development during later stages.

**Table 2:** Lethality rates as larvae and pupae in *Drosophila melanogaster*/*D. santomea* hybrid females

Line	Larvae average survival rate (%)	Pupae average survival rate (%)
A1200.4	81.610	85.556
B1300.11	87.418	87.209
B1300.17	84.655	82.857
B1300.5	89.404	89.150
Bs14.5	93.801	82.621
BS17.1	86.745	93.111
BS17.4	93.927	82.190
C1350.14	87.500	85.714
C1350.15	77.531	93.167
C1350.18	80.404	90.996
CAR1490.6	77.831	87.443
CAR1566.9	88.213	86.825
COST1250.5	73.948	69.798
COST1270.7	86.705	85.357
Field14	79.048	82.183
Field3.9	94.435	85.197
STO7	74.242	66.468
Line 7	89.167	93.386
OBAT1200.5	87.880	91.414
Quija650.22	90.741	95.556
Quija650.39	85.450	87.118
Rain42	91.538	88.384
STO18	81.197	72.639
Thena13	92.726	85.051
Thena3	84.658	94.192
Thena5	93.430	85.859

## Discussion

Previous reports have conclusively demonstrated that in *D. melanogaster*/*sim-group* hybrids, a mitotic effect leads to male lethality during larvae or prepupae stages (Orr et al. 1997; Bolkan et al. 2007)**.** In this report, we demonstrate that there are distinct causes for hybrid male lethality in other *Drosophila* hybrids. Our data show that in *D. melanogaster*/*D. santomea* hybrid males, the defects leading to hybrid inviability arise much earlier in development than in *D. melanogaster*/*sim-group* hybrid males and cause hybrid lethality in embryonic stages. The phenotype observed in the hybrid males is not observed in cuticles of either of the two parental species, which suggests the existence of negative epistasis in hybrids, as alleles that normally function in pure species cause severe developmental defects, leading to hybrid male lethality.

*D. melanogaster*/*D. santomea* hybrid males carry a full set of autosomes of each species, the cytoplasmic elements, mitochondrial genome and maternally deposited genes and the X-chromosome from *D. melanogaster*. The hybrid females carry the same genetic elements but also carry the X-chromosome from *D. santomea*. Thus, several nonexclusive genetic mechanisms could lead to the inviability of hybrid male embryos in *D. melanogaster*/*D. santomea* hybrids. First, it is possible that a recessive antimorphic allele on the *D. melanogaster* X-chromosome interacts with alleles on the *D. santomea* autosomes to cause hybrid male lethality. These effects would not be observed in the hybrid females because the X-chromosome from *D. santomea* masks any recessive effects. Second, it is also possible that a semidominant allele on the *D. melanogaster* X-chromosome causes hybrid inviability in both hybrid males and females, but the presence of a homologous *D. santomea* allele ameliorates the developmental issues in hybrid females, allowing for a partial rescue of viability. Third, it is possible that hybrid inviability is caused by negative epistasis between the Y-chromosome from *D. santomea* and either cytoplasmic elements of *D. melanogaster*, autosomes from *D. melanogaster* or both. Finally, there could be a failure in dosage compensation in hybrid males that leads to hybrid inviability. This possibility, however, seems unlikely as, at low but appreciable frequency, female hybrid embryos also manifest patterning defects or ablations specific to abdominal segments 5–7. Furthermore, forward genetic screens failed to identify any role for dosage compensation defects in male inviability of *D.melanogaster*/*D.simulans* hybrids (Barbash 2010; this of course, does not directly apply to hybrids between *D. melanogaster* and *D. santomea*). This, combined with the variable female viability seen across isofemale lines, suggests that a polygenic epistatic mechanism, and not a failure in dosage compensation, leads to the developmental defects (and subsequent lethality) in *mel*/*san* hybrids. Further research will formally test these hypotheses.

This report, however, is not the first instance in which hybrid embryonic lethality has been observed in *Drosophila*. Presgraves (2003) was able to identify 40 chromosomal regions that lead to hybrid males embryonic lethality. In that case, the nature of the epistatic interactions was between a recessive *D. simulans* autosomal allele and a recessive *D. melanogaster* allele in the X-chromosome. In the *D. melanogaster*/*D. santomea* hybrids, however, the epistatic interaction must be between an allele in the *D. melanogaster* X-chromosome and a dominant or semidominant factor (s) on the autosomes of *D. santomea*. In a second case, the females from the cross *D. simulans* × *D. melanogaster* die as embryos. The identity of the causal allele for this lethality has been precisely established: *Zhr*, a minisatellite in the X*-*chromosome from *D. melanogaster* that causes a viability defect by directly inhibiting chromatid separation in hybrids (Ferree and Barbash 2009). A third case of embryonic lethality is observed in the interspecific hybrids between *D. montana* females to *D. texana* males. In this cross, only male offspring are produced. The genetic mechanism causing hybrid inviability has been ascribed to an incompatibility between the *D. montana* ooplasm and the *D. texana* X-chromosome which causes the lethality of female hybrid embryos before hatching (Kinsey 1967). These last two cases constitute some of the few exceptions to Haldane's rule in *Drosophila*.

Our results also suggest that hatched female embryos are likely to remain viable through subsequent development, as the rate of larval and pupal lethality is low. This, however, does not mean that these female hybrids are fit. In addition to being sterile, with only empty, rudimentary ovaries these females show extensive abdomen sclerotinization. These phenotypes are also observed in *D. melanogaster*/*sim*-group hybrids (Sturtevant 1920). It remains to be determined if the genetic and/or developmental causes for sclerotinization and female sterility are the same in *D. melanogaster*/*D. santomea* and *D. melanogaster*/*sim*-group hybrid females.

This report also demonstrates that there are differences in female viability contingent on the zygotic genome contributed by the *D. santomea* isofemale line. These findings are in keeping with the variability of penetrance and dominance of alleles involved in hybrid inviability of *D. melanogaster*/*D. simulans* interspecific crosses reported by Gérard and Presgraves (2012), demonstrating the critical effects of genetic background of the maternal line on hybrid phenotypes. Their observations show that hybrid inviability has altered penetrance in different backgrounds, suggestive of multiallelic interactions with alleles not fixed in *D. simulans*. Our phenotypic analyses of cuticles reveal that some females do die as embryos, indistinguishable from hybrid males, suggesting that the genetic mechanisms that cause male hybrid embryonic lethality are not exclusively sex-specific, whereas its penetrance is. Modifiers of these genetic mechanisms, however, seem to be segregating in the population, as different isofemale lines show varying amounts of female embryonic lethality. The variability seen in our study, together with the work of Gerard and Presgraves serves as a reminder on the importance of taking into account the highly polygenic nature of the epistatic interactions leading to hybrid inviability.

The fact that we are able to produce hybrids with all assayed *D. santomea* lines demonstrates that the ability of *D. santomea* to cross with *D. melanogaster* is not dependent on a particular mutation in a single line and suggests that any *D. melanogaster* female × *D. santomea* male cross will produce progeny. We have not, however, succeeded in obtaining progeny from the reciprocal cross (*D. santomea* females × *D. melanogaster* males): we have never observed progeny in more than 10,000 crosses of *D. santomea* females with different lines of *D. melanogaster* males. Dissection of *D. santomea* female reproductive tracks in these crosses (*n* > 50,000 attempted females) revealed no sperm in any female, indicating that sexual isolation is complete between these two species in the set of environmental conditions we are applying. Although we will continue attempting these hybridizations, other approaches might be required to produce *D. santomea*/*D. melanogaster* hybrids with *D. santomea* maternal factors. Sanchez and Santamaria (1997), for example, performed transfers of *D. yakuba* (and *D. teissieri*) pole cells into *D. melanogaster oskar* null mutants and were able to produce hybrid progeny (males and females that carry the cytoplasmic elements of *D. yakuba*). This approach, however, does not guarantee that we will obtain *san*/*mel* hybrids as the genetic architecture of hybrid inviability of *yak*/*mel* hybrids may differ from that of *san*/*mel* hybrids.

This study demonstrates how the examination of the genetic basis of hybrid inviability between *D. melanogaster* and *D. santomea*, two relatively diverged species, can shed light on the genetic architecture of cryptic divergence in genes directly involved in the fundamental aspects of body plan formation in *Drosophila*.

## Supporting Information

**Figure S1.** Consistent head involution defects seen in hybrid embryos from different isofemale lines.

**Table S1.** Stocks used in this study.

**Table S2.** Sex ratio in *D. santomea*.
