# Supplementary figures and images for: Embryonic lethality leads to hybrid male inviability in hybrids between Drosophila melanogaster and D. santomea
Source: Ecol Evol. 2013 Apr 23;3(6):1580–9. doi: 10.1002/ece3.573 (PMC3686193; doi:10.1002/ece3.573)

Supplemental Figure 1.

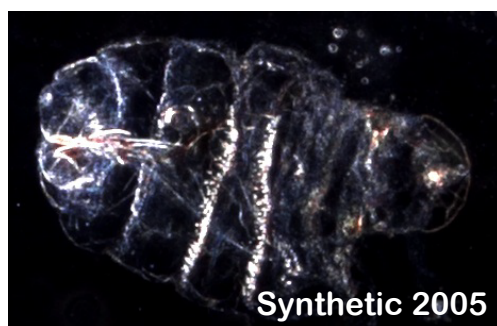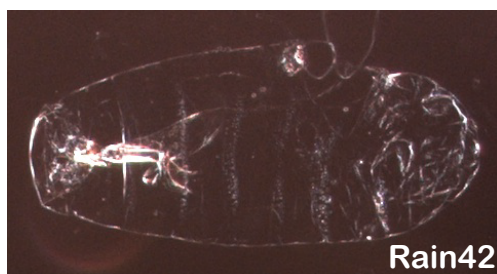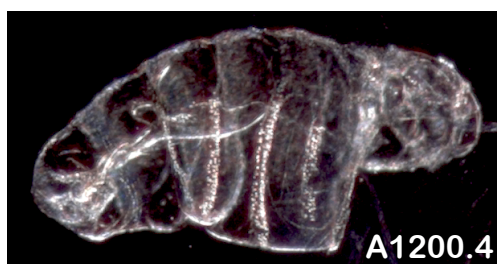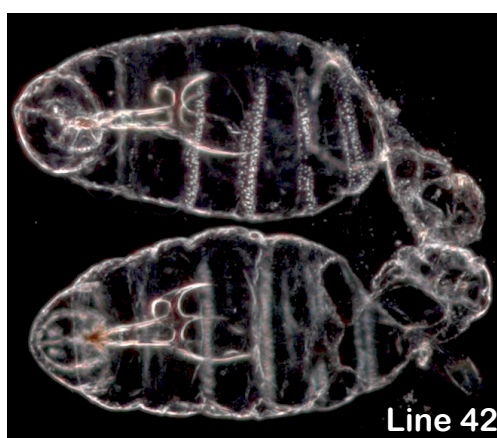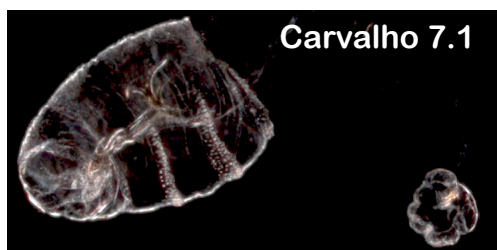

Supplement: Supplementary file 1 [file ece30003-1580-SD1.pdf]
