# Supplementary material for: Embryonic lethality leads to hybrid male inviability in hybrids between Drosophila melanogaster and D. santomea
Source: Ecol Evol. 2013 Apr 23;3(6):1580–9. doi: 10.1002/ece3.573 (PMC3686193; doi:10.1002/ece3.573)
Supplement: Supplementary file 2 [file ece30003-1580-SD2.docx]

**SUPPLEMENTARY TABLE 1.** Stocks used in this study.

| **Species** | **Line** | **Isofemale/ Synthetic** | **Year** | **Collected by:** | **Altitude** |
| --- | --- | --- | --- | --- | --- |
| *D.santomea* | Quija650.22 | ISO | 2005 | Coyne/Lachaise/Llopart | 650 |
| *D.santomea* | A1210.3 | ISO | 2009 | Llopart | 1,210 |
| *D.santomea* | B1300.11 | ISO | 2009 | Matute | 1,300 |
| *D.santomea* | B1300.17 | ISO | 2009 | Matute | 1,300 |
| *D.santomea* | B1300.5 | ISO | 2009 | Matute | 1,300 |
| *D.santomea* | Bs14.5 | ISO | 2009 | Matute | 1,150 |
| *D.santomea* | Bs17.1 | ISO | 2009 | Matute | 1,150 |
| *D.santomea* | Bs17.4 | ISO | 2009 | Matute | 1,150 |
| *D.santomea* | C1350.14 | ISO | 2009 | Matute | 1,350 |
| *D.santomea* | C1350.15 | ISO | 2009 | Matute | 1,350 |
| *D.santomea* | C1350.18 | ISO | 2009 | Matute | 1,350 |
| *D.santomea* | CAR1490.6 | ISO | 2001 | Coyne/Lachaise | 1,490 |
| *D.santomea* | CAR1566.9 | ISO | 2001 | Coyne/Lachaise | 1,566 |
| *D.santomea* | COST1250.5 | ISO | 2009 | Matute | 1,250 |
| *D.santomea* | COST1270.7 | ISO | 2009 | Matute | 1,270 |
| *D.santomea* | Field14 | ISO | 2009 | Matute | 1,250 |
| *D.santomea* | Field3.9 | ISO | 2009 | Matute | 1,250 |
| *D.santomea* | STO7 | ISO | 2001 | Coyne/Lachaise/Llopart | 1,240 |
| *D.santomea* | Line 7 2005 | ISO | 2005 | Coyne/Lachaise/Llopart | 1,300 |
| *D.santomea* | OBAT1200.5 | ISO | 2009 | Matute | 1,200 |
| *D.santomea* | Quija650.22 | ISO | 2005 | Coyne/Lachaise/Llopart | 650 |
| *D.santomea* | Quija650.39 | ISO | 2005 | Coyne/Lachaise/Llopart | 650 |
| *D.santomea* | Rain42 | ISO | 2009 | Llopart | 1,240 |
| *D.santomea* | STO18 | ISO | 2001 | Coyne/Lachaise | 1,240 |
| *D.santomea* | Thena13 | ISO | 2009 | Llopart | 1,300 |
| *D.santomea* | Thena3 | ISO | 2009 | Llopart | 1,300 |
| *D.santomea* | Thena5 | ISO | 2009 | Llopart | 1,300 |

**SUPPLEMENTARY TABLE 2.** Sex ratio in *D. santomea*. P-values show the right-tailed probability of the χ^2^ distribution with df=1.

| **Line** | **Males** | **Females** | **P** |  |  |  | |  |
| --- | --- | --- | --- | --- | --- | --- | --- | --- |
| **A1200.4** | 231 | 256 | 0.257 |  | | |  |  |
| **B1300.11** | 340 | 352 | 0.648 |  | | |  |  |
| **B1300.17** | 287 | 261 | 0.267 |  | | |  |  |
| **B1300.5** | 258 | 274 | 0.488 |  | | |  |  |
| **Bs14.5** | 325 | 318 | 0.783 |  | | |  |  |
| **BS17.1** | 227 | 237 | 0.642 |  | | |  |  |
| **BS17.4** | 273 | 279 | 0.798 |  | | |  |  |
| **C1350.14** | 189 | 201 | 0.543 |  | | |  |  |
| **C1350.15** | 328 | 286 | 0.090 |  | | |  |  |
| **C1350.18** | 251 | 227 | 0.272 |  | | |  |  |
| **CAR1490.6** | 282 | 248 | 0.140 |  | | |  |  |
| **CAR1566.9** | 236 | 241 | 0.819 |  | | |  |  |
| **COST1250.5** | 229 | 224 | 0.814 |  | | |  |  |
| **COST1270.7** | 255 | 222 | 0.131 |  | | |  |  |
| **Field14** | 256 | 290 | 0.146 |  | | |  |  |
| **Field3.9** | 296 | 308 | 0.625 |  | | |  |  |
| **STO7** | 322 | 331 | 0.725 |  | | |  |  |
| **Line 7** | 245 | 253 | 0.720 |  | | |  |  |
| **OBAT1200.5** | 313 | 296 | 0.491 |  | | |  |  |
| **Quija650.22** | 287 | 199 | 0.000 |  | | |  |  |
| **Quija650.39** | 241 | 225 | 0.459 |  | | |  |  |
| **Rain42** | 318 | 306 | 0.631 |  | | |  |  |
| **STO18** | 346 | 332 | 0.591 |  | | |  |  |
| **Thena13** | 301 | 302 | 0.968 |  | | |  |  |
| **Thena3** | 228 | 248 | 0.359 |  | | |  |  |
| **Thena5** | 254 | 276 | 0.339 |  | | |  |  |
